# A Review on the Biological Activity of *Camellia* Species

**DOI:** 10.3390/molecules26082178

**Published:** 2021-04-09

**Authors:** Ana Margarida Teixeira, Clara Sousa

**Affiliations:** 1 LAQV/REQUIMTE, Departamento de Ciências Químicas, Faculdade de Farmácia, Universidade do Porto, 4050-290 Porto, Portugal; ana_margaridart@hotmail.com; 2 CBQF—Centro de Biotecnologia e Química Fina-Laboratório Associado, Escola Superior de Biotecnologia, Universidade Católica Portuguesa, Rua Diogo Botelho 1327, 4169-005 Porto, Portugal

**Keywords:** antimicrobial, antitumor, antifungal, phenolics, flavonoids, ABTS

## Abstract

Medicinal plants have been used since antiquity to cure illnesses and injuries. In the last few decades, natural compounds extracted from plants have garnered the attention of scientists and the *Camellia* species are no exception. Several species and cultivars are widespread in Asia, namely in China, Japan, Vietnam and India, being also identified in western countries like Portugal. Tea and oil are the most valuable and appreciated *Camellia* subproducts extracted from *Camellia sinensis* and *Camellia oleifera*, respectively. The economic impact of these species has boosted the search for additional information about the *Camellia* genus. Many studies can be found in the literature reporting the health benefits of several Camellia species, namely *C. sinensis*, *C. oleifera* and *Camellia japonica*. These species have been highlighted as possessing antimicrobial (antibacterial, antifungal, antiviral) and antitumoral activity and as being a huge source of polyphenols such as the catechins. Particularly, epicatechin (EC), epigallocatechin (EGC), epicatechin-3-gallate (ECG), and specially epigallocatechin-3-gallate (EGCG), the major polyphenols of green tea. This paper presents a detailed review of *Camellia* species’ antioxidant properties and biological activity.

## 1. Introduction

The *Camellia* genus of the family Theaceae is among the most traditional and famous plants in Asia [1], especially in China and Japan. Different species and cultivars are prevalent in many countries across the world, including in Portugal. Some species belonging to *Camellia* genus have a high economic value due to their subproducts such as tea, oil seed, iconic flowering shrubs and ornamental plants [2]. The high abundance and economic value has boosted interest in the biological properties of this plant genus and several studies were undertaken to evaluate its antioxidant, antimicrobial and antitumor activity. Most of the studies on the *Camellia* genus were focused in three species; namely, *C. sinensis* (the tea plant), *C. oleifera* (used to obtain edible oil) and *C. japonica* (ornamental flowers). Tea is the second most consumed beverage worldwide, just after water [3] and is most consumed as black, green or oolong tea, being also found as white, pu’erh or red tea [4,5]. Black tea is preferred in Western countries (78%), followed by green tea (20%) and oolong tea (approximately 2%) [6]. Despite being from the same plant, *C. sinensis* or *C. sinensis* var *assamica* leaves, the different types of teas are obtained according to the fermentation process used. The edaphoclimatic conditions and the agricultural practices strongly impact the composition and properties these plants [6]. Tea leaves are mainly composed by alkaloids, steroids, terpenoids, and polyphenols [7], including catechins [8]. *Camellia* oil is obtained from *C. oleifera* seeds and is one of the four edible tree oils (palm oil, olive oil and coconut oil). It is mainly composed by neutral lipids (88.2%), especially oleic acid (86.3%—*C. japonica;* 67.7–76.7%—*C. oleifera*). and is commonly named as “Asian olive oil” because of the high oleic acid concentration, very similar to that of olive oil [9,10]. After oil extraction, *C. oleifera* seeds still are of interest (the so called *Camellia* seed cake) and have been used over the years as a material for washing hair, for itching and pain relief and to produce cosmetics and food supplements [11]. Over the years a huge amount of works was published demonstrating that these species are an enormous source of antioxidant compounds with proven biological activity against bacteria, fungi and virus as well as antitumor potential [3,11,12,13]. This review aimed to summarize all of these works giving a global and critical perspective of the properties of such an important genus. The antioxidant activity is discussed firstly (Section 2. Antioxidant Properties) and, due to the large amount of available data, the most relevant antioxidant parameters quantified in the literature were summarized in a table (Table 1). The next section is dedicated to the antimicrobial activity and includes antibacterial, antifungal and antiviral properties. The antitumor activity is explored in Section 4 and Section 5 is dedicated to other benefits on health and diseases. Finally, a concluding remarks section was added to stress that despite the large amount of published literature, further additional works still are of relevance, particularly if focused on *Camellia* species other than *C. sinensis*, *C. japonica* and *C. oleifera*.

## 2. Antioxidant Properties

*Camellia* species are unanimously considered a natural source of antioxidant compounds. Most of the studies focus on *C. sinensis*, *C. japonica* and *C. oleifera* being the leaves the best characterized part of the plant through their extracts or purified compounds (Table 1). Extracts were obtained from aqueous or organic solvents (methanol, ethanol, butanol, acetone, ethyl acetate, *n*-hexane, chloroform, acetonitrile, isopropanol) under different experimental conditions of temperature and extraction time. Chromatographic fractions of the extracts are also frequently investigated. *C. sinensis* and *C. sinensis* var assamica seems to be the best characterized species due to the commercial relevance of their leaves. Indeed, the comparison between seeds and leaves extracts (aqueous and organic) as well as their eluted fractions revealed a higher TPC and DPPH scavenging activity in the leaves [14]. Despite the apparently lower antioxidant potential of the seeds, a seed saponin was reported as possessing a significant H_2_O_2_ scavenging activity at different pH values [15]. Beyond the differences in the antioxidant activity reported in the different plant structures, the extraction solvents and the maturity of the plant structure (namely of the leaves) highly impact the results. A study comparing the TPC and DPPH scavenging activity of green and fresh tea, aqueous and organic extracts, eluted with ethyl acetate and water [16] reported higher antioxidant potential of the organic extracts eluted with ethyl acetate for both kinds of leaves. Regarding the leaves maturity, a high antioxidant potential was encountered in fresh leaves. Black, green and white teas were also compared regarding their TPC, TFC, total tannin content (TTC), ABTS and DPPH scavenging activities. Globally, green tea was reported as possessing higher phenolic, flavonoid and tannin content [17,18,19] as well as ABTS scavenging activity [17] than black tea, while white tea seems to possess a middle activity [17]. Among black teas, several differences were also reported in the TPC, TFC and DPPH scavenging activity [19,20] being the results impacted by the extraction solvent [20] as already reported [16]. A 50% organic extraction solvent seems to be able to extract more phenolics than the corresponding absolute solvent [20]. Other studies reported *C. sinensis* as possessing high antioxidant potential when compared with other plant extracts [21,22].

Despite being the same species, *C. sinensis* var assamica, is a quite different plant which possess wider and larger leaves and is frequently found in warmer regions with tropical climates. This variety is frequently pointed as possessing higher polyphenol and lower amino acids content. Several studies reported its antioxidant properties which seems also to be highly impacted by the extraction solvent [7,23,24,25]. The higher TPC and TFC contents seems to be obtained with simple alcohols as methanol and ethanol [7,23] as well as the radical scavenging activity. As reported for *C. sinensis*, the kind [26] of leaves, their maturity [23,27] and the processing methods [24,28] also lead to significant differences in the antioxidant properties of *C. sinensis* var assamica. Despite the low consistency of the results (highly dependent on the solvent used), the TPC seems to be lower for mature leaves [23,27] while the TFC seems to be higher [23]. Differences among the antioxidant potential of the leaves were also correlated with the drying process [28] and with the decaffeination process [24]. Higher TPC, TFC and DPPH scavenging activity were reported for dried leaves (mostly oven dried ones) than for fresh leaves [28]. The decaffeination process seems to decrease the antioxidant activity of the tea [24]. Two additional studies were found in literature focused in soluble and insoluble phenolics [29] and flavanones [30]. Yang and colleagues [29] explored in depth the antioxidant activity of Chinese mistletoes from *C. sinensis* var. assamica identifying eighteen phenolic compounds. Soluble phenolics clearly exhibited higher TPC and TFC and also higher radical scavenging activity. Regarding the work developed by Zhou et al. [30], five novel and seven already known flavanones were identified with high α-glucosidase activity.

Another popular *Camellia* species due to its ornamental flowers, *C. japonica*, was the target of some studies aiming at characterizing its antioxidant potential. Leaves aqueous and alcoholic extracts [31,32,33,34] as well as multiple organic solvent extracts from flowers were investigated [35,36,37]. Aqueous leaves extracts demonstrated antioxidant activity [31] in FRAP and DPPH assays in a dose dependent manner (DPPH assay). The remaining studies including leaves of *C. japonica* were undertaken using organic solvents; however, the different or non-specified maturity of the leaves and cultivars diversity used prevents a direct comparison among the obtained results. Páscoa and colleagues [1] performed a thorough study on the antioxidant properties of this species through its TPC, TFP and ABTS assays comparing around 31 different cultivars from two gardens. These authors concluded that the antioxidant potential of this species is highly dependent on the cultivars studied as well as on the geographic origin, being quite different even among trees of the same cultivar. In this context, care is needed when assigning these properties to a particular species in a general manner. A study comparing mature and green leaves concluded, similarly to the results obtained with *C. sinensis*, that green leaves possess higher scavenging activity [32]. Regarding the results obtained with alcoholic extracts, higher TPC, TFC, superoxide, hydrogen peroxide radical scavenging and ferrous ion chelating activities were obtained with ethanol (an observed trend among other species) while higher total carotenoids and L-ascorbic acid contents were obtained with methanol [33]. Steam plus leaves acetone and methanol extracts were also compared and acetone extract presented a higher activity in the DPPH and β-carotene-linoleic acid assays [34]. Three additional studies [35,36,37] were found in the literature focused on the flowers of this species involving different assays and extraction methodologies. Although no direct comparisons could be performed, all authors concluded that this part of the plant also possesses considerable antioxidant activity deserving additional studies to better characterize it.

*C. oleifera* is a source of cooking oil which has already proven effects on skin diseases and as a burn treatment. Due to its economic relevance, this species was also quite studied regarding its antioxidant properties, particularly its seed oil and cake, the byproduct obtained after the oil refining process. Regarding the oil, several organic compound were used for the extraction steps and a higher radical scavenging activity (DPPH and ABTS assays) was obtained with methanol [38]. Other authors, in a different context, also found a pronounced antioxidant activity of methanolic extracts of the seed oil [39]. Yu and colleagues developed an extraction method based on water and were able to achieve better performance in the ABTS assay with this new method than with an organic solvent [40]. An unconventional work was also developed with and without steam explosion pre-treatment and the authors concluded that the steam explosion increased the DPPH scavenging activity of the extract [41]. Defatted seeds were also investigated in a study which compared the proficiency of isopropanol and butanol as extraction solvents being the best results obtained with isopropanol [42]. Several authors have demonstrated the antioxidant potential of seed cake components, namely of isolated kaempferol glycosides [43], saponins [44], ad purified polysaccharides [45,46] and pomace [47]. Zhu and colleagues compared the impact of two extraction methods (mechanochemical-assisted extraction-MCAE and with heat reflux-HRE) on the DPPH scavenging activity and in the ferric thiocyanate assay of kaempferol glycosides and concluded that the former method led to a better performance [43]. Other studies also concluded that the experimental conditions [44], the extraction method [46] and the solvents [47] all impact the antioxidant activity of the extracts. Indeed, the antioxidant activity of a seed cake saponin seems to be pH dependent (pH = 9.0 being the optimal value) and temperature independent [44]. On the other hand, ethyl acetate extracts possess higher radical scavenging activity when compared with the ones obtained with ethanol and butanol [47]. A unique study was dedicated to the leaves of this species [48]. The authors evaluated the antioxidant potential of three fractions of a crude polysaccharide and concluded that all showed remarkable antioxidant activities in a concentration-dependent manner.

Beyond these three relevant species, *C. sinensis*, *C. japonica* and *C. oleifera*, other *Camellia* species were investigated [49,50,51,52,53,54,55]. However, the few reports found used distinct assays and experimental conditions preventing any comparison of the antioxidant potential of the species. Gao and co-workers [49] evaluated the antioxidant activities of 19 phenolic compounds from *Camellia taliensis* leaves and concluded that they possess significant DPPH radical scavenging activity. Hydrolysable tannins and flavan-3-ols with catechol and/or pyrogallol groups in the molecule presented stronger DPPH radical scavenging activities. Also, other authors reported the DPPH and ABTS scavenging activity of a flavan-3-ol dimer isolated from *C. talensis* leaves [50]. Fruit and seed shell and pomace of *Camellia tenuifloria* organic and aqueous fractions were reported by Chiou [51] as possessing antioxidant activity, being the activity dependent on the part of the plant and solvent considered. Other authors, using different species, obtained similar results in what concerns to the variability of the antioxidant potential of the species with the part of the plant considered [52]. Studies on less common *Camellia* species are also found in literature [53,54,55] stressing the high potential of this plants as a source of bioactive molecules.

## 3. Antimicrobial Activity

The antimicrobial activity of different *Camellia* species was also widely reported; namely, antibacterial, antiviral and antifungal. Similarly, to the antioxidant activity, most of the studies were dedicated to the three species with higher economic impact being *C. sinensis* and its compounds of higher relevance [56,57].

### 3.1. Antibacterial Activity

Antibiotic resistance is a worldwide problem which leads to high costs and deaths, mainly in the hospital context (https://www.ecdc.europa.eu/en/healthcare-associated-infections-acute-care-hospitals/surveillance-disease-data/report, accessed on 12 March 2021). The antibacterial activity of natural products has been largely explored due to the growing antibiotic resistance noticed in several bacterial species [58,59,60,61]. *Camellia* species have been highly explored over the years as a natural source of new compounds with antibiotic activity. Published studies evaluated the activity of several *Camellia* species (*C. sinensis*, *C. sinensis assamica*, *C. japonica*, *C. oleifera*, *Camellia reticulata*, *Camellia sasanqua* and *Camellia semiserrata*) against Gram negative and Gram positive bacteria (Table 2). The popular tea plant species was the most explored one and was reported as possessing activity against multiple bacteria.

One of the first attempts to explore the bacterial activity of *C. sinensis* was undertaken by Yildirim et al. [58]. These authors used water extracts of Rize tea and young shoot tea against *Staphylococcus aureus*, *Candida albicans*, *Escherichia coli*, *Bacillus subtilis* and *Pseudomonas aeruginosa* and, contrary to most of the studies, no antimicrobial activity was detected in the referred extracts. It should be noted that such absence could be related with the extraction procedure. Many studies in several contexts mention that the extraction procedures highly affect the extracted compounds and consequently the biological activity of the extracts. Several years later, Tsai et al. [59] reported the capacity of crude methanolic extracts of green tea to inhibit the growth of several oral pathogens (*Streptococcus mutans*, *Streptococcus sanguinis* and *Streptococcus sobrinus*). In 2009, Erol et al. [16] studied the antibacterial activities of various fresh tea leaves and green tea extracts (ethanol, methanol and water extracts) against *S. aureus*, *Listeria monocytogenes*, *E. coli*, *Hafnia alvei*, *Salmonella enteritidis*, *E. coli* Type 1 and *Bacillus cereus*. Water extracts did not show antibacterial activity for any of the bacteria while ethyl acetate fractions of extracts showed antibacterial activity on *S. aureus* and *B. cereus*. It should be noted that the absence of antimicrobial activity reported for the water extract was in accordance with the findings of Yildirim et al.’s [58] studies reinforcing the relevance of the extraction method. Beyond the extraction procedure, the pH value could also influence the activity of the extract. Indeed, Li et al. [15] proved that the higher the pH value, the worse the antimicrobial activity of tea saponins against Gram-positive bacteria *S. aureus* and Gram-negative bacteria. Of note, the best pH value was an acidic one, namely pH = 4.8. Dhaouadi et al. [17] reported also the antimicrobial activity of the polyphenols of commercialized green and black Tunisian tea decoctions (green/black tea) against Gram positive *S. aureus* and *Staphylococcus epidermidis* and the Gram negative *P. aeruginosa*, *E. coli* and *Salmenolla wiam.* The authors noticed that the inhibition zones were proportional to the phenolic content, suggesting the implication of these molecules in the observed activities. Kiran et al. [60] also proved the relevance of the tea polyphenols. These authors studied the effect of black tea (*C. sinensis*) on virulence traits of clinical isolates of *Shigella dysenteriae* and *E. coli*. The tea-treated cultures showed a decrease of the time necessary for bacterial killing. Indeed, crude tea polyphenols of black tea improved (*p* < 0.05) phagocytic uptake of tea-adapted *S. dysenteriae* cells in 30 min by 37.68% and phagocytic killing by ten-fold in 3 h. Chan et al. [61] studied the antibacterial properties of different brands of green, black and herbal teas of *C. sinensis* against the Gram positive bacteria *Micrococcus luteus, S. aureus*, and *B. cereus*, and Gram negative bacteria *E. coli, Salmonella typhi* and *P. aeruginosa*. The results showed no inhibitory activity of teas against any of the Gram negative bacteria. However, *M. luteus* and *B. cereus*, were inhibited by black tea and herbal teas and all of the Gram positive were inhibited by green tea, being the best results reported for *M. luteus*. The teas were obtained with hot water which validated the inactivity of *C. sinensis* reported by previous studies against Gram negative bacteria; however, this study reported activity against Gram positive ones. Similar results were obtained by Orak et al. [18] regarding the activity of white, green and black tea extracts. Also, Arbia et al. [62] reported antimicrobial activity of aqueous extracts against Gram negative bacteria (*Porphyromonas gingivalis* and *Prevotella intermedia*). These somehow contradictory results obtained by several authors regarding the antimicrobial activity of aqueous extracts need further clarification. Abidi et al. [63] also studied the activity of aqueous extracts of *C. sinensis* against *S. epidermidis*; however, in combination with current antibiotics such as vancomycin, erythromycin, tetracycline, chloramphenicol, ampicillin, ofloxacin, cephalexin and gentamicin. The authors noticed an appreciable synergistic activity between the plant extracts and the tested antibiotics. Granato et al. 2016 [4] studied the characterization of binary and ternary mixtures of green, white and black tea extracts in vitro antibacterial activity against several microorganisms. The results revealed that black and white teas show the highest antimicrobial effects against *Salmonella Typhimurium*; however, the combination of green/white and green/black teas also showed a significant inhibitory effect depending on the considered serotype.

Most of the reported studies were performed with plant leaves; however other plant parts revealed to possess antimicrobial activity. Namely, Rana et al. [64] evaluated the activity of catechins from underutilized tea plant parts (coarse leaves, flowers and fruits) against Gram positive and Gram negative food-borne pathogens (*B. subtilis*, *S. aureus, E. coli* and *Klebsiella pneumoniae*). The best result was obtained for tea flower extract (maximum zone of inhibition at 20 mg/mL) against *S. aureus* and *K. pneumonia*.

Üstündag˘ et al. [65] also discussed the hypothesis of the black tea processing waste (BTPW) being the source of antimicrobial phenolic compounds against *Shigella flexneri* and *B. cereus* and the effects of solvent (water, 50% ethanol and 80% ethanol) on the antimicrobial activity. Water and ethanolic extracts showed antimicrobial activity against all bacteria tested being the best results obtained for aqueous ethanol extracts which possess higher phenolic content.

In 2019, Shetta et al. [66] compared for the first time the antimicrobial activity of raw and green tea essential oils in encapsulated chitosan nanoparticles. The authors found that green tea essential oils nanoparticles possess a greater activity against *S. aureus* with ~9.4 folds’ improvement compared to pure green tea oil and ~4.7 fold against *E. coli*. From this study arises that not only the compounds extraction procedure is a relevant step on the microbial activity but also the way they are used.

Four additional studies were found in literature discussing the antimicrobial activity of *C. sinensis* var *assamica*. Kristanti and Punbusayakul [67] studied the antimicrobial activity of 5 samples of commercial Assam Green Tea against *S. aureus*, *S. typhimurium*, *E. coli* and *L. monocytogenes*. All samples possess higher activity against Gram-positive bacteria being the better results reported in samples with higher total polyphenol content which is in accordance with previously reported studies. Mehrotra et al. [68] also reported antimicrobial activity of this *C. sinensis* variety against *Vibrio cholerae, S. aureus* and *P. aeruginosa*. Abdulbaqi et al. [69] reported a relevant synergistic effect of green tea (*C. sinensis* var. *assamica*) and *Salvadora persica* L. against primary colonizers of dental plaque *Streptococcus mitis*, *S. sanguinis* and *Actinomyces viscosus*. Also, Kawarai et al. [70] studied biofilm formation of *S. mutans* using extracts from Assam tea and green tea. The results revealed that Assam tea (high concentration of galloylated catechins) possess stronger biofilm inhibition activity against than green tea (higher concentrations of galacturonic acid, such as pectin). These studies suggest that *Camellia* extracts may also have potential applications in preventing dental caries, dental plaque control and other oral health problems associated with serious damage.

Also, *C. japonica* was evaluated regarding its antimicrobial activity against several bacteria (*S. typhimurium*, *E. coli*, *L. monocytogenes*, *S. aureus*, *B. subtilis*, *Streptococcus faecalis*, *K. pneumoniae*, *P. aeruginosa* and *S. epidermidis*). Kim et al. [71] describe the antibacterial activity in extracts of *C. japonica* L. petals (extracted with methanol and fractionated into basic, acidic, and neutral fractions). None of the microorganisms was totally inhibited and the basic fraction showed no antibacterial activity. Gram negative *S. typhimurium* and *E. coli* demonstrated slightly greater sensitivity than the Gram positive *L. monocytogenes* and *S. aureus* to the extracts. Moon and Kim [33] studied the antimicrobial activities of fermented *C. japonica* L. leaf extracts (70% ethanol and 100% methanol) against *S. epidermidis*, *B. subtilis*, *K. pneumonia* and *E. coli*. The authors concluded that the ethanol extracts possess higher antimicrobial activities against all bacteria and that the antimicrobial activity was doses dependent for both extracts. Sharma et al. [72] also reported antimicrobial activity in several microorganisms but of gold nanoparticles using *C. japonica* L. leaf extract. The activity seems to be similar for all the tested bacteria.

Three additional studies were found in literature reporting some antimicrobial activity of other Camellia species as *C. oleifera* [54,73]; *C. reticulata* and *C. sasanqua* [54] and *C. semiserrata* [74].

Although most of the studies to evaluate the antimicrobial activity of *Camellia* species extracts were undertaken in vitro, Thakur et al. [13] tested aquoethanolic (1:1) leaves extracts of *C. sinensis* against carbapenem resistant *E. coli*, in vivo, in a murine model (Sprague Dawley rats). The results showed that a single doses extract (5 mg/Kg bwt; oral, +24 h) was very effective against this pathogen. Also, Khan et al. [75] undertook a comprehensive in vitro and in vivo study of the antimicrobial activity of green tea seed extracted and isolated saponins various strains of Gram positive and negative bacteria (*E. coli*, *S. aureus*, and six serovars of *Salmonella: S. typhimurium*, *S. enteritidis*, *Salmonella gallinarum*, *Salmonella choleraesuis*, *Salmonella pullorum* and *Salmonella dublin)* Results showed that the green tea isolated saponins fractions possess antibacterial activity both in in vitro and in vivo experiments.

The antimicrobial activity of *Camellia* species extracts was mainly explored against human pathogens. A single study was found in literature that reports the activity of green tea extracts in *Pseudomonas syringae* pv. actinidiae (Psa), the agent that causes kiwifruit bacterial cancer [76]. The results showed that tea extracts strongly inhibited Psa growth and swimming motility. Also, a significant loss of virulence was observed after tea extract treatment.

**Table 2:** Antibacterial activity of *Camellia* species.

Species	Extracts/Pure Compounds	Bacterial Species	Antibacterial Activity	Ref.
*C. sinensis*	Dried leaves Black teas (rize tea and young shoot tea)	*S. aureus*, *B. subtilis*, *E. coli*, *P. aeruginosa*	ND	[58]
	Green tea dried leaves	*S. mutans*, *S. sanguinis* and *S. sobrinus*	MIC: >8, 4 and >8 (mg/mL), respectively.	[59]
	Green tea leaves (MetOH, EtOH, H_2_O extracts and their crude, ethyl acetate and water fractions)	*S. aureus*, *B. cereus*, *L. monocytogenes*, *E. coli*, *S. enteritidis*, *H. alvei*	Detected for ethyl acetate fraction for *S. aureus* and *B. cereus*	[16]
	Phenolic extracts form green and black tea leaves infusions	*S. aureus*, *S. epidermidis*, *E. coli*, *P. aeruginosa* and *S. wiam*	*S. aureus* and *S. epidermidis* were the most sensitive tested organisms, and decoction time and method affected antimicrobial activity.	[17]
	Black and green tea leaves	*M. luteus*, *S. aureus*, *B. cereus*, *E. coli*, *S. typhi*, *P. aeruginosa*.	ND activity against the three Gram-negative bacteria. Green teas inhibited all three Gram-positive bacteria with *S. aureus* being the least susceptible. Black teas inhibited the growth of *M. luteus* and *B. cereus*, but not *S. aureus*.	[61]
	White, green and black tea (leaf powder)	*S. aureus*, *E. coli* and *S. enteritidis*.	Detected activity against *S. aureus.*	[18]
			ND against *E. coli* and *Salmonella* enteritidis.	
			Green tea demonstrated the best results.	
	Dried leaves aqueous extracts	*S. epidermidis*	Detected additive and synergistic antibacterial activity with antibiotics (highest with erythromycin and cephalexin) against *S. epidermidis*.	[63]
	Leaves H_2_O-EtOH extract	Carbapenem Resistant *E. coli*	D	[13]
	Leaf, flower and fruit tea cathecins	*B. subtilis*, *S. aureus*, *E. coli*, *K. pneumoniae*	Detected for all photogenes with a minimal concentration of 10 mg/mL.	[64]
	H_2_O and H_2_O-EtOH extracts of black tea and black tea waste	*S. aureus*, *B. cereus*, *S. flexneri*	Detected for all bacterias with aqueous ethanol extracts significantly higher.	[65]
	Green tea leaves	*P. gingivalis*, *P. intermedia*.	D	[62]
	Gunpowder green tea	*P. syringae pv. actinidiae*	D	[76]
	Green tea saponins from seeds	*S. aureus*, *E. coli*, 6 *Salmonella* serovars	D	[75]
	Tea saponins seeds	*S. aureus*, *E. coli*	Detected for both species. Lower pH’s displayed highest activity	[15]
	Oil	*S. aureus*, *E. coli*	D	[66]
*C. sinensis* var *sinensis*	Green, white and black tea lyophilized infusion	*B. cereus*, *S. Typhimurium*, *S. aureus*, *P. aeruginosa*, *E. coli*, *Salmonella*	D	[4]
*C. sinensis* var *assamica*	Green tea infusion	*S. aureus*, *L. monocytogenes*, *S. typhimurium*, *E. coli*	All teas inhibited gram-positive better than gram-negative bacteria.	[67]
	Assam tea leaves	*S. aureus*, *V. cholera*, *P. aeruginosa*	D	[68]
	Green tea powder leaves	*S. mitis*, *S. sanguinis*, *A. viscosus*	Synergistic anti-plaque effect between *C. sinensis* var. *assamica* and *S. persica* L.	[69]
*C. sinensis* var *assamica*, *C. sinensis*	Assam tea and Green tea leaves	*S. mutans*	Assam tea has stronger biofilm inhibition activity against *S. mutans*	[70]
*C. japonica*	Petals MetOH and H_2_O extracts	*L. monocytogenes*, *S. aureus*, *S. typhimurium*, *E. coli*	Gram-negative demonstrated greater inhibition than the gram-positive.	[71]
	Fermented leaves MetOH and EtOH extracts	*S. epdermidis*, *B. subtilis*, *K. pneumoniae*, *E. coli*	Ethanol extracts exhibited higher antimicrobial activity	[33]
	Encapsulated leaves in gold nanoparticles	*B. subtilis*, *S. aureus*, *S. faecalis*, *K. pneumoniae*, *P. aeruginosa*, *E. coli*	D	[72]
*C. oleifera*, *C. reticulata C. sasanqua*	Seeds virgin Oils	*B. cereus*, *E. coli*	D	[54]

ND—not detected; D—detected; MIC—minimum inhibitory concentration.

### 3.2. Antifungal Activity

The antifungal activity of *Camellia* spp was evaluated by several authors, being most of the studies focused on the popular plant tea, *C. sinensis*. Fungus and yeasts were studied and the reported results were not always consensual. The heterogeneity of the results seems not to be related with the use of crude extracts versus purified compounds once antifungal activity was reported using both. Nevertheless, the works showing no *Camellia* antifungal activity only used crude extracts.

Rize tea and young shoot tea, two Turkish black tea extracts, were reported as ineffective against *C. albicans*, one of the most opportunistic pathogens, as well as against other microorganisms [58]. Also, Chakraborty and Chakraborti in 2010 [77] concluded that the leaves methanolic extract of green tea (*C. sinensis*) has no activity against two species of *Aspergillus*. Both studies included four distinct extracts concentrations which somehow reinforce their conclusions.

Despite these two works, *Camellia* species have been pointed as a great source of bioactive compounds including antifungal ones. *C. albicans* seems to be susceptible to several catechins (pH dependent), which also enhance the antifungal effect of amphotericin B and fluconazole [78]. Also, Li et al. [15] reported a pH-dependent activity of commercial tea saponins (isolated from *C. sinensis* seeds) against *C. albicans*. In this study, low pH values lead to lower minimum inhibitory concentrations while in the Hirasawa and Takada [78] work, acidic conditions decreased the effectiveness of the considered catechins. Choi et al. [14] also studied the antifungal activity of green tea seeds using, however, homemade extracts. The study evaluated three different fractions: F1—non-adsorption fraction; F2—eluted with 40% methanol and F3—eluted 100% methanol against *C. albicans, Cryptococcus neoformans, Alternaria alternate* and *Rhizoctonia solani* cultures. The results shown a positive correlation between the concentration and inhibitory activity being fractions F2 and F3 the most active.

The previous studies reporting the antifungal activity were performed using somehow purified compounds; however, other works also report such activity using crude extracts. Archana and Abraham in 2011 [79] did a comparative analysis of the antifungal activity of leaf extracts from fresh green tea, commercial green tea and black tea on *Fusarium, Aspergillus fumigatus, Aspergillus niger* and *C. albicans.* The fresh green tea methanolic extract was found to have higher activity. Later, Orak et al. [18] also studied the antifungal activities of tea extracts (white, green and black). All extracts exhibited antifungal activity against two aflatoxigenic moulds *Aspergillus parasiticus* with a positive correlation with the extract concentration. The results obtained by Orak and colleagues were in agreement with those obtained by Archana and Abraham in 2011 [79] being the green tea extracts the ones presenting the higher inhibitory rates.

The popularity of *C. sinensis* due to their highly appreciated subproduct, tea, totally eclipses the remaining *Camellia* species and few studies were dedicated to them. Feás et al. 2013 [54] studied the activity of seeds virgin oils from *C. oleifera, C. reticulata* and *C. sasanqua* against clinical strains of *C. albicans*. The three oils evidenced antifungal activity exhibiting different MICs. *C. reticulata* and *C*. *oleifera* oils showed the best and similar antifungal activity (20.833 ± 7.217 mg/mL) followed by (20.833 ± 7.217 mg/mL) *C. sasanqua* (29.167 ± 19.094 mg/mL). In 2015, Meng et al. [74] studied the antifungal activity of the crude extractum from *C. semiserrata* Chi (Nanshancha) seed cake (NSC) against three postharvest pathogens (*Colletotrichum musae*, *Colletotrichum gloeosporioides* and *Penicillium italicum*) performing in vitro and in vivo (Fruit Test) tests. The in vitro tests showed a higher inhibition rate for *C. musae*, followed by *C*. *gloeosporioides* and *P. italicum.* Such values were quite similar to those obtained with tea saponin for *C. musae* and *P. italicum* being even higher for *C*. *gloeosporioides* (90.9 ± 1.7% versus 49.8 ± 3.2%). The in vivo antifungal activity was evaluated by means of the fruit lesion caused by the three fungi after exposition to NSC crude extractum. The extractum proved to be able to eliminate the lesion diameter in banana; to kept it constant and lower when compared with the untreated fruit for mango and to maintain it slightly lower for the Shatang mandarin when compared with the untreated fruit and tea saponin effect.

### 3.3. Antiviral Activity

Flavonoids are a group of phenolic compounds present in plants mostly as secondary metabolites being widespread in their flowers, fruits (including nuts) or roots as well as in their sub products like wine or tea. Indeed, tea is pointed as an innate immunity modulator which can enhance immune response in order to mitigate COVID-19 (SARS-COV-2) [80]. Its phenolics are believed to possess several benefits on health and can act as antiviral agents being their efficiency against human immune deficiency virus (HIV) [81], Herpes simplex virus, influenza virus [82] and hepatitis B and C virus [83] already reported. Frequently, flavonoids need to be concentrated or structurally modified to enhance their antiviral ability which can justify the few studies reporting this property in *Camellia* species (published literature is summarized in Table 3).

Epigallocatechin-gallate (EGCG) is the major flavonoid present in green tea and seems to possess a highly antiviral potential through several action mechanisms. (EGCG was able to inhibit the binding of the HIV-1 glycoprotein 120 to the CD4 molecule on T cells [84] and to prevent HIV-1 infection [85]. Yamaguchi et al. [86] also studied the inhibitory effects of EGCG on the life cycle of HIV-1 and its mechanisms of action in T-Lymphoid and monocytoid cell systems and concluded that EGCG interact in many steps of the HIV-1 life cycle. Liu et al. [87] proved the efficacy of EGCG from green tea against HIV-1 but reported a higher activity of EGCG when obtained from black tea. EGCG proved also to be able to inactivate adenovirus [88] and Epstein-Barr virus [89].

Influenza viruses were also the target of several studies using *Camellia* species extracts or pure compounds. In 2005, Song et al. [90] studied the antiviral effect of green tea catechins on influenza virus and demonstrated that the EGCG and ECG ((−)-epicatechin gallate) were potent influenza virus replication inhibitors in Madin-Darby canine kidney (MDCK) cell cultures. This effect was observed in all influenza virus subtypes tested, including A/H1N1, A/H3N2 and B virus and attributed to the 3-galloyl group of the catechin skeleton that seems to be able to alter the physical properties of viral membrane. The same viruses were studied later by other authors [91] which concluded that theaflavin derivatives are anti-influenza compounds through the inhibition of the viral HA gene replication. The inhibitory effect of EGCG on the growth of Influenza A and B viruses in MDCK cells was also studied by Nakayama et al. [92] and Imanishi et al. [93]. The first authors reported that EGCG agglutinated influenza viruses and prevented the viruses from adsorbing to MDCK cells while Imanishi and colleagues noticed that GTE exerts an additional inhibitory effect on the acidification of intracellular compartments such as endosomes, lysosomes and thereby inhibiting the growth of both viruses. A more complete study evaluated the activity of several tea polyphenols against influenza viruses A and B. The authors [94] reported the inhibitory activity of five and six polyphenols against influenza A and B, respectively and stablished a structure-activity relationship. Generally, dimeric molecules such as theaflavin and procyanidin B-2 displayed more potent activity than monomeric ones and that the planar structure of kaempferol is crucial for the anti-influenza B activity. *C. sinensis* raw extracts (black tea) also demonstrated activity against Influenza H5N1, a highly pathogenic avian influenza virus, inoculated in MDCK cells [95]. Indeed, Baatartsogt and colleagues [95] compared the activity of several row extracts from commercial teas and found that hibiscus and black tea were effective in virus titers reduction. A more daily application against influenza virus was reported by Shin et al. [96] which demonstrated that the virus infectivity on the skin cell layer became obsolete when washed with a green tea solution.

Tea also demonstrated activity against herpes simplex viruses type 1 and 2. Prodelphinidin B-2 3′-O-gallate) isolated from green tea leaves was reported by Cheng et al. [97] as possessing anti-herpes simplex virus type 2 (HSV-2) activity (IC_50_ = 5.0 ± 1.0 µM and 1.6 ± 0.3 µM for XTT and plaque reduction assays, respectively). Prodelphinidin B-2 3′-O-gallate seems affected the late stage of HSV-2 infection and also shown to inhibit the virus from attaching and penetrating into the cell. Oliveira et al. [98] demonstrated the activity of three theaflavins isolated from black tea against herpes simplex type 1 in Vero and A549 cells. The authors claim the relevance of their study based on the growing herpes simplex virus resistant strains and the need of developing new antiherpesviral treatments. Also, other common tea catechin derivatives demonstrated to possess antiherpetic activity [99].

In 1998, Clark et al. [100] studied the in vitro capacity of theaflavins extracted from black tea to neutralize bovine rotavirus (NCDV-Lincoln strain to BSC-1 cell line) and bovine coronavirus (BCV ATCC P2 to HRT-18 cell line) infections. Both compounds proved to have inactivation capacity of the two studied virus.

**Table 3:** Antiviral activity of *Camellia* species.

Species	Extracts/Compounds	Viruses	Activity	Ref.
*C. sinensis*	EGCG	HIV-1	HIV-1 binding inhibition	[84]
			Infection prevention	[85]
			Affects HIV-1 life cycle	[86]
		Adenovirus	Virus inactivation	[88]
		Epstein-Barr	Virus inactivation	[89]
		Influenza A and B	Virus aglutination	[92]
			intracellular compartments acidification	[93]
	EGCG, ECG	Influenza (A/H1N1, A/H3N2, B)	Replication inhibition	[90]
	Theaflavins	Influenza A and B	Viral HA gene replication inhibition	[91]
		HSV-1	Virus inhibition	[98]
		Bovine rotavirus	Virus inactivation	[100]
	Tea polyphenols	Influenza A and B	Virus inhibition	[94]
	Tea raw extract	Avian influenza (H5N1)	Virus titers reduction	[95]
	Prodelphinidin B-2 3′-O-gallate	HSV-2	Attaching and penetration cell inhibition	[97]

## 4. Antitumor Activity

Okuda et al. [101] was one of the first authors to report the antitumor activity of *Camellia* spp. namely, *C. oleifera* and *C. sinensis*. Epigallocatechin gallate (EGCG), the main polyphenol of green tea, was referenced as a “cancer preventing agent”. Later, many other studies proved the antitumor activity of *Camellia* spp being most of them associated with *C. sinensis*. The antitumor potential of this species was highly explored whether using raw *C. sinensis* leaves extracts, their purified compounds or their commercially available polyphenols alone or synergistically with the most used antitumor drugs.

In 1999, Suganuma et al. [102] studied the synergistic effects of (2)-epicatechin (EC) with (2)-epigallocatechin gallate (EGCG); (2)-epigallocatechin (EGC) and (2)-epicatechin gallate (ECG) in the inhibition of lung cancer cell line PC-9 growth. Their studies were motivated by discovering that green tea extract (tea itself) has stronger inhibition effect in the tumor cells growth than the polyphenol content would have indicated. Indeed, the authors proved that the apoptosis of PC-9 cells was increased synergistically by the use of two polyphenols and by the use of EGCG in a cotreatment with sulindac (more pronounced effect) and tamoxifen. In 2000, Mimoto et al. [103] evaluated the effect of EGCG on cisplatin-induced lung tumours in A/J mice. Cisplatin is used to treat lung cancer but is also able to induce cancer in animal models. Based on that, the authors evaluated the EGCG; cisplatin and EGCG plus cisplatin activity. Tumour multiplicity (5.1 ± 2.1 to 2.8 ± 2.3) and cisplatin induced weight loss (24.7–26.3% to 10.8–11.6%) decreased when cisplatin and cisplatin plus EGCG treatments were compared. Also, Sartippour et al. [104], evaluated the synergistic effect of green tea and tamoxifen, however against breast cancer. The authors observed that green tea enhanced the inhibitory effect of tamoxifen on the proliferation of the ER (estrogen receptor)-positive MCF-7, ZR75, T47D human breast cancer cells increasing also their apoptosis in vitro. The in vivo results were also promising; mice treated with the combination demonstrated the smallest MCF-7 xenograft tumor size and also the highest levels of apoptosis.

Despite the synergistic antitumor effects of tea polyphenols, mostly EGCG, noticed by the previous reported studies, their potential was also evaluated alone. Mittal et al. [12], dedicated their studies to the breast cancer cells (MCF7-line) and reported an EGCG dose-dependency inhibition of the colony forming potential (20–100%) and a cell viability decreases (around 80%) with no adverse effect on the growth of normal cells. The decrease of cell viability was associated with the inhibition of telomerase activity (40–55%), mRNA expression (40–55%) and protein expression of hTERT (human telomerase reverse transcriptase). Roy et al. [105], achieved quite similar results to those obtained by Mittal and colleagues but using the breast cancer cell line MDA-MB-468. These authors reported an EGCG dose-dependent inhibition of cell proliferation (15–100%) and cell viability decrease (3–78%) also associated to apoptosis increase. The activity of green tea EGCG was also investigated against ovarian carcinoma cell lines (p53 negative, SKOV-3 cells; mutant type p53, OVCAR-3 cells; and wild type p53, PA-1 cells), by Kim et al. [106]. Similar to previous studies, the antitumor effect was highly doses dependent and via apoptosis induction and cell cycle arrest which varies with the cell line considered. Recently, saponins from tea seed pomace of *C. oleifera* were also reported as possessing cytotoxic activity on MCF-7 cells [107]. Saponins were fractioned under specific chromatographic conditions and the fractions obtained revealed differential antitumor activity. The antitumor activity of the raw *Camellia* extracts was also evaluated against distinct tumour cell lines. Extracts were obtained were obtained from different species (*C. sinensis*, *C. oleifera* and *C. japonica*) and different parts of the plant. Choi et al. [14] studied the in vitro biological activity of several fractions of green tea seeds at two concentrations (50 µL/mL and 100 µL/mL). The antitumor activity was evaluated against three different cell lines: HEC-1B (endometrial adenocarcinoma); HEP-2 (larynx carcinoma) and SK-OV-3 (ovary carcinoma). Fractions were non-eluted (F1) and eluted with 40% (F2) or 100% (F3) of methanol. Despite the higher antioxidant activity founded in F1, the higher antitumor activity was reported in the fraction eluted with 100% of methanol (F3). In 2010, Carvalho et al. [108] studied for the first time the effect of green tea (raw leaves extracts) against renal cell carcinoma lines A-498 and 769-P. The antiproliferative activity of the methanolic extract was examined and the results showed a powerful growth inhibition of both cell lines in a concentration-dependent manner (IC_50_ = 54 ± 10 for A-498 and 129 ± 28 µg/mL for 769-P cells) It should be noted that, along with the antitumor activity studies, the authors identified the extract phenolic and methylxanthine content allowing a deeper knowledge on the compounds responsible for the reported biological activity. In 2016, Rana et al. [64], evaluated the bioactivity (including the antitumor against oral cancer cells) of frequently neglected tea plant parts as apical buds, stem, coarse leaves, flowers, fruits and roots. The results showed that tea fruit extract exhibited higher toxicity (71.6 to 74.9% at the concentration of 50–200 µg/mL) against oral cancer cells being comparable to the obtained with standard vinblastibe (74.2% at 2 µg/mL concentration). A positive splenocytes proliferation in mice within 24 h was also observed. Curiously, the extracts from flowers and leaves neither showed significant toxicity nor proliferation of splenocytes. The major tea constituents in different parts of the plant were also identified.

Regarding *C. oleifera*, in 2012, Jin reported the antitumor activity of the polysaccharide obtained from seed cake and fruit shell of *C. oleifera* Abel in two different studies [109,110]. An antitumor rate of 85.6% was obtained (doses dependent) against Sarcoma 180 solid tumour grown in mice with seed cake polysaccharide and of 65.2% with seed shells. The authors claim that such compound possess “powerful tumor-fighting properties in vivo,” without relevant cytotoxicity. Also, the few and inexpensive extraction and isolation steps of the polysaccharide could be an added advantage for industry. Tingting Li et al. [111] reported the in vitro antitumor activities of the three major ingredients (saponin, protein, and polysaccharide) obtained from defatted seeds against the human hepatoma cell line (Hep G2 cells) and normal rat liver cell lines (IAR20). Despite the remarkable antitumor activity exhibited by the three compounds, better results were obtained with the polysaccharide against Hep G2 cells (IC_50_ = 5.826 µg/mL), followed by saponin (IC_50_ = 26.754 µg/mL) and protein (IC_50_ = 36.794 µg/mL)The inhibition rate of the three compounds on Hep G2 cells was higher than 80% at the higher concentration tested (200 µg/mL) being on IAR20 cells dependent on the considered compound (1.35 ± 0.07, 0.61 ± 0.03 and 25.27 ± 0.09% for protein, polysaccharide and saponin, respectively). Later, Chaikul et al. [112] studied the cytotoxicity and activity on melanogenesis of *C. oleifera* seed oil with the ultimate goal of utilize it as a functional oil in health, cosmetic products and feed. Cytotoxicity was evaluated by the sulforhodamine B (SRB) assay in B16-F10 melanoma cells and 3T3-L1 cells demonstrating cell viabilities of 94.59 ± 3.41% and 97.57 ± 1.62%, respectively. The melanogenesis revealed to be via inhibition of tyrosinase and tyrosinase-related protein-2 activities. According to the authors, their results clearly indicated the potential of the seed oil as a functional oil.

*C. japonica* is a *Camellia* species commonly used as an ornamental plant and is often overlooked regarding its potential as a bioactive compound source, due to the popularity of *C. sinensis* and *C. oleifera*. A single study, regarding its antitumor activity performed by Thao et al. in 2010 [113] was found in literature. These authors evaluated the in vitro cytotoxic activity against the cancer cell lines A549, LLC, HL-60, and MCF-7 using the MTT assay method of *C. japonica* triterpenoids obtained from stem barks. Three newly identified compounds along with seven known ones were tested. From them, the newly identified 3β-*O*-acetyl-16β-hydroxyolean-12-en presented cytotoxicity against LLC and HL-60 cancer cell lines (IC_50_ = 25.2 and 21.7 mM, respectively). The remaining compounds shown weak or moderate cytotoxic activity against some of the tested cell lines. An additional study was found reporting the antitumor activity of a very uncommon camellia species, *Camellia osmantha* [114]. The authors reported strong inhibitory activity against bladder cancer T-24 cells of ethanolic extracts obtained from the core fruit of this species.

Lastly, an uncommon study was undertaken by Chitsazan in 2015 [3] to prove the anti-cancer properties of four type catechins of green tea (epicatechin; epigallocatechin; epicatechin-3-gallate and EGCG) via quantum mechanics calculations, namely ab initio method. The author also proposed a relation between the biological activity and the chemical structure of the catechins.

## 5. (Other) Benefits on Health and Diseases

Medicinal plants have been used since antiquity exhibiting high benefits on health and proved pharmacological activities. Several species among *Camellia* genus have been pointed as possessing a set of benefits including anti-osteoporotic, anti-inflammatory and antidiabetic activity, as blood pressure and body fat reducing agents among others (Table 4). Negishi et al. [115] evaluated black and green tea polyphenols’ capacity to attenuate blood pressure increases in stroke-prone spontaneously hypertensive rats. The results shown that during the daytime, systolic and diastolic blood pressure were considerably lower in the black and green tea polyphenol groups than in control groups. Also, in 2007, Potenza et al. [116] studied the EGCG benefits in endothelial function, insulin sensitivity and reduction of blood pressure against myocardial I/R injury in spontaneously hypertensive rats (SHR) and conclude that EGCG therapy was able to simultaneously improve metabolic and cardiovascular pathophysiology in SHR, to reduce infarct size and improve cardiac function. Wu et al. [117] demonstrated an anti-osteoporotic effect of EtOH extracts of the seeds of *C. semiserrata* Chi., in a tretinoin-induced osteoporotic in vivo rat model and a positive estrogenic activity positively related with the number of sugar moieties and acetyl groups of the extracts. The antidiabetic activity of green tea (Thea sinensis L.) was explored by Miura et al. [118] in genetically type 2 diabetic mice (KK-Ay) whose showed that a dose of 100 mg/Kg body weight of green tea cold extract could reduce the blood glucose 4 and 8 weeks after repeated administration in hyperinsulinemia mices but not in normal mices. In 2018, Zhang et al. [119] also studied the in vitro hypoglycemic activity of polysaccharides from *C. oleifera* seed cake measuring glucose uptake in cultured HepG2 cells and concluded that the extracts can stimulate the consumption of the glucose in the medium by the cells. In 2007, Nagao et al. [120] studied the reduction of body fat and cardiovascular risks in humans by green tea extracts. The study included Japanese women and men with visceral fat-type obesity. After a 2-week diet run-in period, a 12-week double blind parallel multicenter trial which the subjects ingested green tea containing 583 mg of catechins (catechin group) or 96 mg of catechins (control group) per day was performed. The results showed that catechin group decreased in systolic blood pressure, low-density lipoprotein (LDL) cholesterol, body weight and fat mass, index waist and hip circumference and also subcutaneous and visceral fat area. Several years later, Li et al. 2016 [121] studied the impacts of *Camellia kucha* and its main chemical components also on the lipid accumulation in 3T3-L1 adipocytes but in rats. Kucha tea (KT) revealed anti-adipogenic activity (dose-dependent) demonstrating significant decrease in lipid droplet accumulation. KT also diminished the mRNA and protein levels of fatty acid synthase, fatty acid translocase, steroylcoenzyme A desaturase-1, lipoprotein lipase and acetyl-CoA carboxylase-1. Chan et al. 2010 [122] studied the effects of EGCG on retinal pigment epithelial (RPE) cell migration and adhesion. The results demonstrated that EGCG can inhibit cell migration in a dose-dependent manner and RPE cell adhesion to fibronectin preventing epiretinal membrane formation and protect for some eyes diseases. Ye et al. [11] studied the anti-inflammatory and analgesic activities of the hydrolysed sasanquasaponins from the defatted seeds of *C. oleifera*. The results showed anti-inflammatory and analgesic activities with production of pro-inflammatory cytokines and PGE2 inhibited. Levites et al. 2003 [123] studied the neuroprotection and neurorescue against Aβ toxicity and PKC-dependent release of non-amyloidogenic soluble precursor protein by green tea polyphenol EGCG. The authors concluded that EGCG can protect and rescue PC12 cells against the β-amyloid (Aβ) toxicity (in a dose-dependent manner). Yoon et al. [124] studied the anti-hyperuricemic effect in vitro and in vivo (mice with 4 weeks old) of the biologically active constituents of ethanol *C. japonica* leaf extracts (ECJL). In vitro, ECJL inhibited xanthine oxidase activity by 41.3 ± 5.5% (dose-dependent) and in vivo, at the doses of 100 and 300 mg/kg, inhibited hepatic xanthine oxidase activity, highly attenuating hyperuricemia. Jeon et al. 2018 [125] evaluated the effects of the extracts from fruit and stem of *C. japonica* on induced pluripotency and wound healing in vivo mouse wound model and proved that the extracts enhanced mouse and human induced pluripotent stem cell (iPSC) generation and promoted effective wound healing. Wang et al. [126] reported the protective effects of camellia oil (*Camellia brevistyla*) against indomethacin-induced gastrointestinal mucosal damage in vitro and in vivo (human intestinal Int-407 cells and a mouse model of indomethacin-induced gastric mucosal damage). The experiments revealed that a pre-treatment with Camellia oil can increased cell viability, wound healing and reduced reactive oxygen species production in the cells. In vivo results demonstrated that pre-administration of camellia oil prevented gastric wound damage by decreasing inflammatory mediators as interleukin-6, tumor necrosis factor-α and cyclooxygenase-2 levels. Kim et al. [127] studied the protective effects of *C. japonica* flower extract (CJFE) against urban air pollutants by urban-induced reactive oxygen species (ROS) production in vitro cultured normal human dermal fibroblasts (NHDFs) and in an ex vivo model. CJFE was able to stop the urban air pollutants-induced ROS generation and matrixmetalloproteinase-1 (MMP-1) production and demonstrated very good protective activity against pollutants-induced deteriorating effects in the ex vivo model by reducing the lipid peroxidation marker and the level of pollutants-induced malondialdehyde (MDA). Zhang et al. [128] evaluated the mutagenicity and safety of the water extract of *C. oleifera* Abel (WECO) and find no toxic effects or significant differences on clinical chemistry value, body weight, hematology value or organ/body weight ratio. The NOAEL for WECO was 2 g/kg/BW for subacute toxicity study. These studies included different parts of different *Camellia* species demonstrating the importance of the genus *Camellia* in research for its benefits on health and diseases. Wang et al. [129] evaluated the toxicological effect of dietary excess of saccharicterpenin, the extract of *camellia* seed meal (*C. oleifera*), in piglets. The dietary supplementation of about 500 mg·kg^−1^ had beneficial effects on piglets improving liver glutathione peroxidase (GSH-Px) activity but higher supplementations (as 2500 or 5000 mg·kg^−1^) could lead to growth retardation, hematological abnormalities and organ injuries.

## 6. Concluding Remarks

The antioxidant properties and biological activity of *Camellia* species have been extensively studied over the years, being most of the works focused in the three species with higher economic value, namely, *C. sinensis*, *C. oleifera* and *C. japonica*. It should be stressed that few studies have reported differences among cultivars of the same species which is known to highly impact the biological properties of the plants. Also, no comparisons were found between distinct sample collecting seasons nor geographic regions, other known relevant features. Furthermore, the differences encountered in the presentation of the results prevent easy comparisons of the results of the diverse studies. These gaps may justify additional studies on distinct *Camellia* species that have been somehow neglected, as well as further works comparing the impact of cultivars, collecting seasons and geographic regions on the plant properties. Also, and of high relevance, the already proven biological activity among *Camellia* genus, clearly show that these species should be further considered in future developments of new natural based drugs.

## Figures and Tables

**Table 1:** Antioxidant properties of *Camellia* species.

Species	Extracts/Compounds	Methods *	Range **	Ref.
*C. sinensis*	Seeds (S) and Leaves (L) aqueous extracts and eluted fractions F1-F3	TPC	18.9(S-F1)-25.0(S-F3)-26.2(S)-141.8(S-F2)-234.3(L) mg GAE/g	[14]
		DPPH	93.3(S-F2)-70.3(L)-15.9(S-F3)-15.8(S)-14.8(S-F1) %	
	Seeds tea saponin	H_2_O_2_ scavenging	27.6 ± 1.4(pH = 4.8)//8.9 ± 0.4(H_2_O)//20.5 ± 1.0(pH = 8.0) %	[15]
	Leaves extracts (MetOH/EtOH; H_2_O) of Green (G) and Fresh (F) tea and eluted fractions: Ethyl Acetate-F1;Water-F2	TPC	G:481.8 ± 9.48(MetF1)//560.8 ± 11.99(EtOHF1)//70.2 ± 3.68(H_2_OF1) mg GAE/g ^§^	[16]
			F:523.7 ± 31.92(MetF1)//680.2 ± 4.44(EtOHF1)//615.8 ± 5.98(H_2_OF1) mg GAE/g ^§^	
		DPPH	G:70.8 ± 2.28(MetF1)//64.8 ± 1.76(EtOHF1)//78.6 ± 1.66(H_2_OF1) 100 g AEAA/g ^§^	
			F:74.1 ± 2.19(MetF1)//79.3 ± 0.92(EtOHF1)//80.8 ± 0.13(H_2_OF1) 100 g AEAA/g ^§^	
	Black (B) and Green (G) tea	TPC	B:99 ± 11 to 953 ± 40//G:218 ± 20 to 1388 ± 52 mg GA/100 mL ^§§^	[17]
		ABTS	B:499 to 3637//G:977 to 4975 mg Trolox/100 mL ^§§^	
	Black (B), Green (G) and White (W) tea	TPC	B:161.8 ± 0.73//G:313.3 ± 1.41//W:245.3 ± 1.41 μg GAE/mg	[18]
		TFC	B:15.91 ± 0.06//G:16.98 ± 0.27//W:12.57 ± 0.19 μg QE/mg	
		TTC	B:34.38 ± 0.44//G:266.792.59//W:211.14 ± 1.34 μg TA/mg	
	Black (BOP-broken orange pekoe, FBOP-flowery BOP, RD-red dust); Green(G) tea	TPC	G:26.33 ± 1.73//BOP:8.84 ± 0.5//FBOP:6.78 ± 0.55//RD:8.20 ± 0.49 mg GAE/g	[19]
		TFC	G:50.12 ± 0.60//BOP:17.7 ± 0.82//FBOP:13.93 ± 1.08//RD:19.12 ± 0.33 mg CAT/g	
	Black tea extracts: E1-H_2_O; E2-MetOH; E3-EtOH; E4-EtOH (50%); E5-Acetone; E6-Acetone (50%); E7-EthylAcetate	TPC	E1~7.5//E2~4.5//E3~2.5//E4~7//E5~2//E6~8//E7~15 g/100 g GAE	[20]
		DPPH	E1:0.042 ± 0.001//E2:0.009 ± 0.003//E3:0.05 ± 0.010//E4:0.014 ± 0.003//E5:0.064 ± 0.020//E6:0.003 ± 0.040//E7:0.047 ± 0.010 g/mL	
	Tea bag samples	TPC	1034.48 ± 416.24 mg GAE/L	[21]
		DPPH	68.60 ± 22.40%	
		FRAP	10,331.19 ± 4802.91 μM TEAC/L	
	Dried leaves extracts	TPC	3.5 ± 0.6 μM	[22]
		DPPH	1.93 ± 0.03 mg/mL	
		ABTS	10.70 ± 1.87 μg/mL	
*C. sinensis* var assamica	Green tea extracts: E1-MetOH; E2-Acetone; E3-H_2_O	TPC	E1:18.32 ± 0.357//E2:0.79 ± 0.020//E3:2.62 ± 0.10 mg GAE/g	[7]
		TFC	E1:16.25 ± 0.030//E2:28.75 ± 0.010//E3:17.05 ± 0.007 mg CAT/g	
		DPPH	E1:75.30 ± 0.011//E2:75.00 ± 0.053//E3:80.10 ± 0.003%	
	EtOH-E1, Acetone-E2 and H_2_O-E3 leaves extracts: Tea Shots (TS), Young (Y), Mature (M)	TPC	E1-TS:65.26 ± 0.92//E2-Y:49.89 ± 0.67//E2-M:39.35 ± 2.13 mg GAE/g ^§^	[23]
		TFC	E1-TS:36.36 ± 19.28//E1-Y:34.30 ± 29.88//E2-M:57.30 ± 16.68 mg QE/g ^§^	
		DPPH	E1-TS:31.39 ± 3.04//E3-Y:30.18 ± 1.76//E2-M:25.40 ± 1.21 mg TEAC/g ^§^	
		ABTS	E3-TS:36.87 ± 0.13//E3-Y:36.93 ± 0.09//E2-M:36.80 ± 0.04 mg TEAC/g ^§^	
		FRAP	E2-TS:1.698 ± 0.014//E1-Y:1.751 ± 0.065//E2-M:1.938 ± 0.067 mg TEAC/g ^§^	
	Leaves Decaffeinated (D) and Caffeinated (C) Green tea Extracts (E1-hot H_2_O and E2-EthylAcetate) ^#^	TPC	E1:123.4 ± 13.13(D);143.8 ± 7.19(C)//E2:128.47 ± 7.23(D);185.5 ± 7.57(C) mg GAE/g	[24]
		TFC	E1:14.87 ± 0.39(D);29.3 ± 1.26(C)//E2:10.62 ± 0.53(D);43.5 ± 2.1(C) mg QE/g	
		DPPH	E1:996.1 ± 19.12(D);1403.07 ± 60.13(C)//E2:1124.2 ± 13.5(D);1449.7 ± 72.4(C) mM TEAC/g	
		FRAP	E1:1165 ± 31.2(D);1587.1 ± 79.35(C)//E2:1141 ± 41.2(D);1623.4 ± 81.17(C) mM TE/g	
	Green tea extracts: W-water and R-resin and corresponding fractions (F1,F2)	TPC	326.55 ± 3.21(W)//551.12 ± 5.24(R)//659.83 ± 1.71(F1)//669.55 ± 4.74(F2) mg GAE/g	[25]
		TFC	556.82 ± 26.48(W)//782.37 ± 9.79(R)//974.22 ± 5.31(F1)//960.15 ± 5.87(F2) mg RE/g	
		DPPH	4.71 ± 0.15(W)//4.57 ± 0.09(R)//12.25 ± 1.76(F1)//4.13 ± 0.70(F2) μg/mL	
		HO• scavenging	2.15 ± 0.13(W)//1.81 ± 0.40(R)//1.58 ± 0.24(F1)//1.44 ± 0.08(F2) mg/mL	
	Purple leaves of Zijuan tea	DPPH	~65–82%	[26]
		ABTS	~6–18%	
		FRAP	~60–190 μmol/L	
		CAA	~18–24 μmol QE/100mg	
	Tea shoots (TS), Mature (M) leaves, Green tea (G)	TPC	260.07 ± 13.10(TS)//219.31 ± 19.89(M)//246.62 ± 1.36(G) mgGAE/gDW	[27]
		FRAP	3714.93 ± 48.12(TS)//3001.85 ± 51.7(M)//3171.07 ± 13.83(G) μmol TEAC/gDW	
		ORAC	3950.08 ± 98.15(TS)//3202.65 ± 50.26(M)//3074.84 ± 17.11(G) μmol TEAC/gDW	
	Green tea	TPC	50.79 (fresh leaves) to 209.17 (oven 60 °C) mg/g ^§§^	[28]
		TFC	14.30(microwave) to 38.18(oven 100 °C) mg/g ^§§^	
		DPPH	167.17(oven 60 °C) to 505.50(microwave) μg/mL ^§§^	
	Mistletoes Soluble (SP) and Insoluble-Bound (IBP) Phenolics	TPC	8.65–9.91(SP)//3.95–4.59(IBP) μmol FAE/g	[29]
		TFC	0.93–3.05(SP)//0.10–0.30(IBP) μmol CAT/g	
		FRAP	42.25 ± 1.49(SP)//8.07 ± 0.75(IBP) μmol FAE/g	
		H_2_O_2_ scavenging	1429.34 ± 7.69(SP)//1383.79 ± 3.33 μmol FAE/g	
		DPPH	2.19 ± 0.11(SP)//1.51 ± 0.07(IBP) μmol FAE/g	
		ABTS	81.03 ± 0.90(SP)//5.78 ± 1.24(IBP) μmol TEAC/g	
	Leaves 12 C-geranylated flavanones from YingDe black tea	DPPH	6.2 ± 0.07 to >200 μg/mL ^§§^	[30]
		α-glucosidase	10.2 ± 0.04 to 89.7 ± 0.09 μM ^§§^	
*C. japonica*	Leaves H_2_O extract (Extract concentration)	DPPH	37.9 (125 μg/mL)//63.1(250 μg/mL)//91.9(500 μg/mL)//92.2(1000 μg/mL) %	[31]
		FRAP	0.95(EC = 1000 μg/mL)	
	Leaves etanolic extracts	TPC	22.4 ± 1.0 to 147.9 ± 2.9 mg GAE/g dry leaf ^§§^	[1]
		TFC	10.4 ± 1.1 to 75.1 ± 4.1 mg CAT/g dry leaf ^§§^	
		ABTS	18.7 ± 0.6 to 93.1 ± 2.3 mM TEAC/g dry leaf ^§§^	
	Leaves 1,3-butylene glycol extracts: Mature-F1 and Green leaves-F2	H_2_O_2_ scavenging	F1 = 3.489 ± 0.623//F2 = 0.878 ± 0.152 mg/mL	[32]
		HO• scavenging	F1 = 0.163 ± 0.008//F2 = 0.079 ± 0.007 mg/mL	
	Leaves methanol-E1 and ethanol-E2 fermentad extracts	TPC	E1 = 27791 ± 336//E2 = 32274 ± 240 mg GAE/100 g	[33]
		TFC	E1 = 19,273 ± 416//E2 = 20519 ± 291 mg RE/100 g	
		TCC	E1 = 1711 ± 24//E2 = 1586 ± 15 mg/100 g	
		AAC	E1 = 491 ± 31//E2 = 258 ± 25 mg AA/100 g	
		DPPH	E1 = 0.23 ± 0.004//E2 = 0.22 ± 0.003 mg/mL	
		Superoxide	E1 = 0.33 ± 0.03//E2 = 0.23 ± 0.02 mg/mL	
		H_2_O_2_	E1 = 0.36 ± 0.01//E2 = 0.28 ± 0.01 mg/mL	
		NO	E1 = 0.35 ± 0.01//E2 = 0.35 ± 0.01 mg/mL	
	Stems + leaves acetone-E1 and MetOH-E2 extracts	DPPH	E1 = 246.56//E2 = 320.17 μg/mL	[34]
		β-carotene-linoleic acid	E1 = 258.19//E2 = 396.88 μg/mL	
	Flower extracts fractions: H_2_O-F1, EtOAc-F2, BuOH-F3, CHCl_3_-F4, n-hexane-F5	DPPH	F1 = 34.5//F2 = 18.0//F3 = 73.2//F4 = >250//F5 = > 250 μg/mL	[35]
	Flower EtOH extracts (Concentration)	DPPH	28(6.25 μg/mL)//49(12.5 μg/mL)//58(25 μg/mL)//60(50 μg/mL) %	[36]
	Flower extracts (organic solvents and hydrolitic enzymes)	TPC	56.7–107.6 mg GAE/g	[37]
		DPPH	410.8–768.6 μmol TEAC/g	
*C. oleifera*	Seed oil extracts: MetOH-E1; acetone-E2; ethyl acetate-E3; acetonitrile-E4	DPPH	E1:66.50 ± 1.08//E2:4.46 ± 1.17//E3:5.92 ± 1.62//E4:0.4 ± 0.96%	[38]
		ABTS	E1:59.21 ± 4.72//E2:10.42 ± 3.25//E3:12.53 ± 3.24//E4:2.32 ± 0.75 µM	
	Seed oil MetOH extract-E1 and Fractions: Benzene-F1; Ether-F2; EtOAc-F3; n-BuOH-F4; Water-F5	TPC	E1:79.5 ± 10.2 mg of p-hydroxybenzoic acid equivalent/kg oil	[39]
		TFC	E1:8.98 ± 0.54 mg of rutin equivalent/kg oil	
		DPPH	E1:52.37 ± 6.5//F1:28.41 ± 2.2//F2:17.42 ± 1.7//F3:43.17 ± 3.2/F4:283.11 ± 12.7//F5:676.73 ± 23.1 µg/mL	
	Seed oil aqueous and organic extract	DPPH	Aqueous: 2.27 ± 0.05//Organic: 3.31 ± 0.07 mg/mL	[40]
	Seed oil: with/without steam explosion	DPPH	With:24.65 ± 1.23//Without:29.74 ± 1.09 mg/mL	[41]
	Defatted sedds extracts: Isopropanol-E1; Butanol-E2	ABTS	E1:287.5 ± 7.5//E2:285.0 ± 2.5 mg/g	[42]
		ORAC	E1:446.7 ± 5.2//E2:53.4 ± 4.7 mg/g	
	Seed cake kaempferol glycosides	DPPH	MCAE:0.0850//HRE:0.1012 mg/mL	[43]
	Seed cake saponins	DPPH	3866 ± 3 µg/mL	[44]
		Phentriol oxidation	4744 ± 2 µg/mL	
		Metal chelating	2389 ± 2 µg/mL	
	Seed cake purified polysaccharides: COP-H, COP-U, COP-E, COP-A	DPPH	COP-H = 0.37//COP-U = 0.45//COP-E = 0.32//COP-A = 0.42 mg/mL	[45]
	Seed cake polysaccharides: COP-1, COP-2, COP-3, COP-4, COP-c	DPPH	COP-1 = 3.35//COP-2 = 0.94//COP-3 = 1.28//COP-4 = 3.67//COP-c = 1.55 mg/mL	[46]
		HO•scavenging	COP-1 = 1.25//COP-2 = 0.79//COP-3 = 1.11//COP-4 = 3.32//COP-c = 2.58 mg/mL	
	Seed cake pomace EtOH extract-E1 and fractions: AcOEt-F1; BuOH-F2	DPPH	E1 = 9.613 ± 0.521//F1 = 0.287 ± 0.032//F2 = 2.029 ± 0.232µg/mL	[47]
	Leaves polysaccharides fractions: F1,F2,F3	Iron chelating	F1 = 3.19//F2 = 2.21//F3 = 2.15 mg/mL	[48]
		DPPH	F1 = 1.69//F2 = 0.86//F3 = 1.27 mg/mL	
		HO• scavenging	F1 = 3.68//F2 = 1.29//F3 = 2.80 mg/mL	
*C. taliensis*	Phenolic compunds from leaves	DPPH	8.2 ± 0.9 to 203 ± 1 μM ^§§^	[49]
	Leaf flavan-3-ol dimer	DPPH	3.0 ± 0.1μM	[50]
		ABTS	21.2 ± 0.9μM	
*C. tenuifloria*	Fruit-F, Seed-S and Pomace-P extract-E and MetOH-M, ButOH-B and H2O-A fractions	TPC	FE:107.37 ± 3.54//FM:266.79 ± 1.85//FB:129.13 ± 2.55//FA:51.85 ± 3.16 mgGAE/g	[51]
			SE:91.42 ± 1.47//SM:266.30 ± 7.29//SB:106.95 ± 3.09//SA:88.90 ± 5.71 mgGAE/g PE:62.40 ± 3.26//PM:120.56 ± 2.16//PB:43.34 ± 0.27//PA:6.26 ± 1.6 mgGAE/g	
		DPPH	FE:19.74 ± 0.19//FM:7.34 ± 0.89//FB:13.18 ± 0.75//FA:27.25 ± 1.30 μg/mL	
			SE:14.30 ± 1.01//SM:5.47 ± 0.28//SB:15.55 ± 0.10//SA:5.82 ± 0.09 μg/mL PE:84.96 ± 2.75//PM:14.38 ± 0.23//PB:170.99 ± 16.69//PA:218.03 ± 23.12 μg/mL	
*C. vietnamensis C. polyodontia C. octopetala C. meiocarpa C. semiserrata C. chekiangoleosa C. oleífera*	Free-FP, Conjugated-CP and Insoluble-Bound-IBP phenolic acids from Seeds: Kernel-K and Shell-S	TPC	*C. oleifera*:14.40 ± 0.10FP-K//*C. semiserrata*:10.35 ± 0.08FP-S// *C. vietnamensis*:11.78 ± 0.09CP-K;11.24 ± 0.08IBP-K //*C. meiocarpa*:7.59 ± 0.05CP-S//*C. chekiangoleosa*:9.28 ± 0.10IBP-S mg GAE/g ^§^	[52]
		ABTS	*C. octopetala*:45.21 ± 6.66FP-K// *C. semiserrata*:69.61 ± 8.79FP-S; 32.57 ± 5.65CP-K//*C. meiocarpa*:43.16 ± 7.2CP-S//*C. vietnamensis*:89.35 ± 4.37IBP-K//*C. chekiangoleosa*:54.21 ± 8.64IBP-S % ^§^	
		FRAP	*C. meiocarpa*:3.83 ± 0.12FP-K//*C. semiserrata*:2.75 ± 0.27FP-S//*C. vietnamensis*:3.74 ± 0.18CP-K;2.43 ± 0.24CP-S;3.36 ± 0.28IBP-S//*C. chekiangoleosa*:3.36 ± 0.33IBP-K ×10 μmol TEAC kg^−1^FW ^§^	
*C. fangchengensis*	Leaf favan-3-ol dimer	DPPH	32.0 ± 0.5 μM	[53]
		ABTS	109.3 ± 4.9 μM	
*C. reticulata C, oleifera C. sasanqua*	Virgin oils	DPPH	*C. reticulata*:33.48 ± 7.65//*C. oleifera*:35.20 ± 4.95//*C. sasanqua*:54.87 ± 8.78 μg/mL	[54]
*C. crassicolumna*	Phenolic compunds from leaves	DPPH	8.9 ± 0.4 to 1039 ± 49 μM ^§§^	[55]

* TPC—total phenolic content by Folin-Ciocalteu assay; DPPH—2,2-diphenyl-1-picrylhydrazyl, free radical scavenging activity; ABTS—2,2-azino-bis(3-ethylbenzothiazoline-6-sulfonic acid; TFC—total flavonoid content; TTC—total tannin content; FRAP—ferric reducing antioxidant power; CAA—cellular antioxidant activity; ORAC-oxygen radical absorbance capacity. ** GAE—gallic acid equivalent; AEAA—ascorbic acid equivalent; QE—quercetin; TA—tannic acid; TEAC—trolox equivalent antioxidant activity; FAE—ferrulic acid equivalents; RE—rutin equivalent; AA—L-ascorbic acid; MCAE—Mechanochemical-assisted extraction; HRE—heat reflux extraction. ^#^ the higher values among 4 cultivars/varieties; ^§^ only the higher values were presented; ^§§^ lower and higher value-range.

**Table 4:** General benefits on health and diseases of *Camellia* species (miscellaneous).

Species	Extracts/Compounds	Activity	Ref.
*C. sinensis*	EGCG	Systolic and diastolic pressure decrease in rats	[115]
		Infarct size reduction and improved cardiac function in rats	[116]
		Inhibition of cell migration and eyes diseases protection	[122]
		Protection and rescue of PC12 cells against the β-amyloid toxicity	[123]
	Green tea leaves extracts	Reduction of blood glucose in hyperinsulinemia rats	[118]
		Decrease in systolic blood pressure, LDL, body weight and fat mass, index waist and hip size in men and women	[120]
*C. semiserrata*	Seeds EtOH extracts	Anti-osteoporotic effect	[117]
	Hydrolysed sasanquasaponins from the defatted seeds	anti-inflammatory and analgesic activities with production of pro-inflammatory cytokines	[11]
*C. oleifera*	Seed cake polyssacharides	Increase of glucose uptake by HepG2 cells	[119]
	Saccharicterpenin	Improvement of liver glutathione peroxidase till 500 mg.kg^−1^	[129]
*C. kucha*	Leaves extracts	Decrease in lipid droplet accumulation	[121]
*C. japonica*	Leaves extracts	Inhibition of xanthine oxidase activity in vitro and in vivo	[124]
	Fruit an steam extracts	Induction of pluripotent stem cell generation and promotion of effective wound healing in mouse and human	[125]
	Flower extracts	Extracts were able to stop urban air pollutants-induced ROS generation and matrixmetalloproteinase-1	[127]
*C. brevistyla*	Oil	Protective effect against indomethacin induced gastrointestinal mucosal damage in vitro and in vivo	[126]

## Sample Availability

Samples of the compounds are not available from the authors.
